# Ancestry-related distribution of Runs of homozygosity and functional variants in Qatari population

**DOI:** 10.1186/s12863-022-01087-1

**Published:** 2022-09-21

**Authors:** Massimo Mezzavilla, Massimiliano Cocca, Pierpaolo Maisano Delser, Ramin Badii, Fatemeh Abbaszadeh, Khalid Abdul Hadi, Girotto Giorgia, Paolo Gasparini

**Affiliations:** 1 Institute for Maternal, and Child Health - IRCCS “Burlo Garofolo”, Via dell’Istria 65/1, 34137 Trieste, Italy; 2 Department of Biology, University of Padua, Padua, Italy; 3 Department of Zoology, University of Cambridge, Cambridge, England; 4 Molecular Genetics Laboratory, Laboratory of Medicine and Pathology, Hamad Medical Corporation (HMC), Doha, Qatar; 5 Audiology and Balance Unit, National Program for Early Detection of Hearing Loss, Hamad Medical Corporation (HMC), Doha, WH Qatar; 6 Department of Surgical, Medical and Health Sciences, University of Trieste, 34149 Trieste, Italy

**Keywords:** Qatar population, Admixture, Runs of homozygosity, Positive selection, Loss of function, Warfarin response

## Abstract

**Background:**

Describing how genetic history shapes the pattern of medically relevant variants could improve the understanding of how specific loci interact with each other and affect diseases and traits prevalence. The Qatari population is characterized by a complex history of admixture and substructure, and the study of its population genomic features would provide valuable insights into the genetic landscape of functional variants. Here, we analyzed the genomic variation of 186 newly-genotyped healthy individuals from the Qatari peninsula.

**Results:**

We discovered an intricate genetic structure using ancestry related analyses. In particular, the presence of three different clusters, Cluster 1, Cluster 2 and Cluster 3 (with Near Eastern, South Asian and African ancestry, respectively), was detected with an additional fourth one (Cluster 4) with East Asian ancestry. These subpopulations show differences in the distribution of runs of homozygosity (ROH) and admixture events in the past, ranging from 40 to 5 generations ago. This complex genetic history led to a peculiar pattern of functional markers under positive selection, differentiated in shared signals and private signals. Interestingly we found several signatures of shared selection on SNPs in the *FADS2* gene, hinting at a possible common evolutionary link to dietary intake. Among the private signals, we found enrichment for markers associated with HDL and LDL for Cluster 1(Near Eastern ancestry) and Cluster 3 (South Asian ancestry) and height and blood traits for Cluster 2 (African ancestry).

The differences in genetic history among these populations also resulted in the different frequency distribution of putative loss of function variants. For example, homozygous carriers for rs2884737, a variant linked to an anticoagulant drug (warfarin) response, are mainly represented by individuals with predominant Bedouin ancestry (risk allele frequency G at 0.48).

**Conclusions:**

We provided a detailed catalogue of the different ancestral pattern in the Qatari population highlighting differences and similarities in the distribution of selected variants and putative loss of functions. Finally, these results would provide useful guidance for assessing genetic risk factors linked to consanguinity and genetic ancestry.

**Supplementary Information:**

The online version contains supplementary material available at 10.1186/s12863-022-01087-1.

## Background

Qatar has a rich and fascinating history, inhabited by humans for approximately 50,000 years with a substantial influx of Arab tribes from the surrounding region, mainly from the Nejd desert to the West. Islam began to flourish in Qatar in the seventh century CE, and the area became an important cultural centre for the spread of the Islamic religion [1]. Like many other Gulf region countries, the Qatari population is affected mainly by diabetes, obesity, and cardiovascular diseases [2], in particular The prevalence of obesity in Qatar is among the highest in the world, 41.4% based on reports from the Qatari Ministry of Public Health (https://phs.moph.gov.qa/data/healthy-lifestyle/), in addition the level of CVD related deaths in Qatar is high as in other high income countries” (https://phs.moph.gov.qa/data/cardiovascular-diseases/).

Thus, it is an interesting “laboratory” to investigate the genetics and environmental risk factors underlying such diseases.

As a matter of fact, genetic disorders are generally well-described by purifying selection models, while complex-disease susceptibility is tied, at least in part, to evolutionary adaptations and demography. In particular, reducing effective population size due to inbreeding and bottlenecks reduces the effectiveness of both positive and purifying selection [3]. The type of selection and the strength of its coefficient vary across populations, affecting the prevalence of causative variants for diseases and traits [4].

Previous data on the Qatari population demonstrated a peculiar clustering and different variance in homozygosity regions (ROH) [5]. Recent data show that ROH across genomes could impact different phenotype distributions across different ancestries [6, 7]. Such changes in the genomic architecture of a given population could also impact the effect of the same variants in different populations. For example, although PPARγ gene variants are associated with diabetes in some individuals of European descent, mutations in this gene were found not to be a risk factor in the Qatari population [8].

In addition, a recent study showed that European-derived polygenic scores (PGS) had reduced predictive performance in the Qatari population [9].

In fact, several studies investigated the pattern of genetic diseases in conjunction with endogamy and consanguinity in the populations of this geographical area [10–12].

An essential piece of information needed is the knowledge of the genetic history and the evolutionary mechanism behind the genomic makeup of the Qatar population. A recent work studied several thousands of individuals highlighted the link with ancient hunter-gatherers and Neolithic farmers from the Levant [13]. However, in our work we aimed to integrate several pieces of information coming from population genetics analyses and we tried to integrate them in order to understand the pattern of deleterious variation in a group of Qatari individuals.

Here, we investigated the genetic structure of 186 newly genotyped individuals from Qatar and analyzed the distribution of ROH regions under recent natural selection and putative loss of function variants.

Our work aims to address the following questions: i) How genetic structure and demography affect the ROH pattern in the Qatari population and ii) how genetic structure affects the pattern of genes under putative positive selection and the distribution of deleterious variants with a specific focus on the loss of function variants. Our final goal is to provide a detailed insight into the genetic makeup of the Qatari population to better estimate and understand the genetic risk factors based on ancestry components, demography and natural selection.

## Results

### Uniparental markers analysis

High variation was observed for mitochondrial DNA (haplotype diversity = 0.873) in both the entire dataset and the subset, including male individuals only. Major haplogroups are represented by H (South West Asia origin), L (Africa origin) and J (Western Asia origin). The Y chromosome shows a reduced diversity with a major haplogroup (J1*) representing 75% of the Y chromosomes analysed (see Fig. S1 A-B-C). The ratio of Y chromosome haplotype diversity (haplotype diversity = 0.574) on mitochondrial haplotype diversity is 0.65.

### Population structure and admixture

**Fig. 1:**
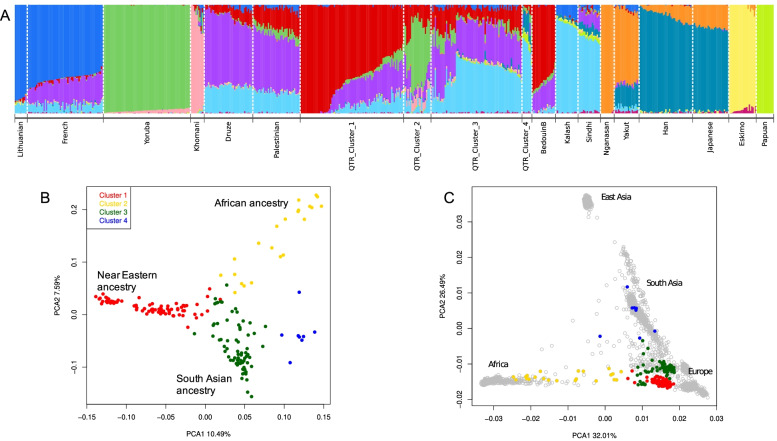
Population structure of Qatar (**A**) Admixture plot for K = 11 using Human Origins dataset. The red colour represents the Middle Eastern-Bedouin like ancestry, the cyan colour represents the South Asian component the violet component represent the Middle Eastern-Palestinian like component, the green one represents the African component and the blue one represents the East Asian component (**B**) Principal component analysis followed by Gaussian clustering based only on 186 Qatari sample, a total of four clusters were found. **C** Projected principal component of 186 Qatari sample onto 1000G populations. We can observe how Cluster 2 shows African ancestry, Cluster 3 has South Asian Ancestry and Middle Eastern Ancestry, Cluster 1 has the highest Bedouin like ancestry and finally Cluster 4 shows evidence of South Asian and east Asian ancestry.

An unsupervised analysis with ADMIXTURE v.1.3 [14] was performed on the Qatari samples using a subset of reference population from the Human Origins dataset downloaded from https://reich.hms.harvard.edu/allen-ancient-dna-resource-aadr-downloadable-genotypes-present-day-and-ancient-dna-data and the lowest cross-validation error was obtained with a total number of cluster equal to 11 (see Table S1. Four major ancestral components differently distributed among individuals were detected (Fig. 1A). The red component was found mainly in the Bedouin population. The green component (found mainly in Yoruba samples) was appreciable only in a fraction of the Qatari sample. The violet (Palestinian) and azure (South Asian) components were found in another group of Qatari individuals showing low levels of both red (Bedouin) and green (African) components. A small group of individuals shows an admixture pattern that contains only South Asian and East Asian ancestry but neither Palestinian nor Bedouin. A full representation of all cluster solution is shown in Fig. S2.

Using the first six principal components from the Principal component analyses (PCA), a gaussian clustering using the approach implemented in Mclust [15] was carried out. An overall number of four clusters was detected: Cluster 1 (red), Cluster 2 (gold), Cluster 3 (green) and finally Cluster 4 (blue), which contains a small fraction of East Asian ancestry (Fig. 1B, Figs. S3-S4).

PCA using as reference the 1000 Genome Project [16] data shows that the Qatari individuals are placed between the European and South Asian pole of variation. Cluster 2 (gold) is spread towards African samples while individuals from Cluster 4 show similar variation to East Asian samples, confirming the ADMIXTURE analysis (Fig. 1C).

**Fig. 2:**
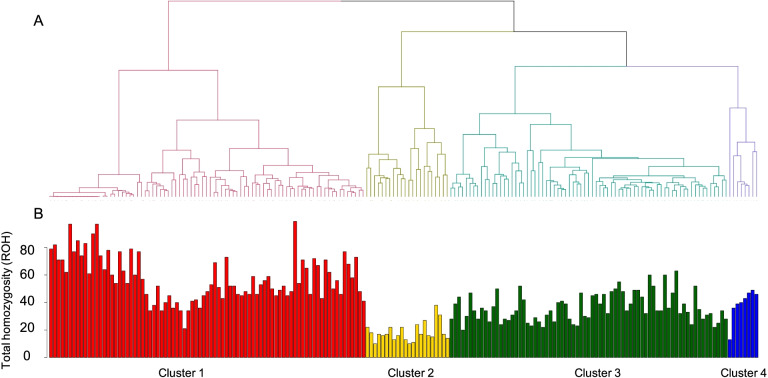
Clustering based on admixture Q values and ROH pattern. **A** dendrogram clustering and **B** each bar represents the individual total homozygosity due to ROH in Mb. Cluster 1 is colored in red, Cluster 2 in yellow, Cluster 4 in dark green and Cluster 4 in blue

In order to better investigate the genetic relationships between individuals using admixture patterns, we used the individual ancestry values obtained from previous admixture analyses to build a distance matrix which was used to generate a dendrogram (Fig. 2A). Each individual was coloured according to their cluster assignment, and for each of them, the level of homozygosity due to ROH (Runs of homozygosity) was collected. As shown in Fig. 2B**,** individuals from Cluster 1 and Cluster 3 show the highest level of ROH. These clusters are characterized by Bedouin and Palestinian/South Asian ancestry. On the other hand, individuals from Cluster 2 (characterized by the highest level of African ancestry) shows the lowest level of runs of homozygosity in our dataset. Interestingly, individuals from Cluster 4 (with both South Asian and East Asian ancestry) show a homozygosity level similar to that of Cluster 3.

**Fig. 3:**
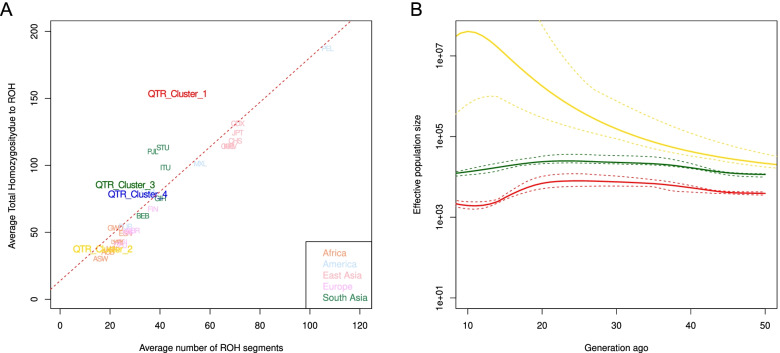
**A **Average level of total homozygosity and number of ROH segments in Qatari population. On the y-axis the ROH were measured in Mb. Population from the 1000G are coloured according to their geographic origin.** B** Effective population size (Ne) estimates with IBDNe. We estimated the Ne of the three major clusters found in the Qatari samples. Dotted lines represent the 95% CI

We also found a sizeable ancestry-related variation in the number of ROH segments and total homozygosity due to ROH when the three clusters were compared to the 1000 Genome populations (Fig. 3A, Fig. S5). We should note that Cluster 1 and Cluster 3 have increased total homozygosity with respect to the average number of segments, which suggests recent consanguinity [17, 18]

Such a diverse distribution could be explained by the different genetic history of each cluster.

Analyses of effective population size (Ne) in the last 50 generations using IBDNe [19] further support a significant difference in the level of genetic drift, as the confidence intervals of the effective population sizes across generations never overlap between each other (Fig. 3B).

Admixture analysis using MALDER [20] revealed several admixture events that happened at different times: one admixture event between 32 ± 3 generations ago for Cluster 1 (in which the reference populations with the highest Zscore, according to MALDER are Greek and Yoruba) and a more recent event 5 ± 0.5 generations for Cluster 2 (reference populations with the highest Z score, according to MALDER are Biaka and Greek).

Interestingly, Cluster 3 shows evidence of two admixture events: one at 42 ± 5 generations ago (reference populations: Greek and Yoruba) and one more recently at 5 ± 1 generation ago (reference populations: Biaka and French). We had to exclude Cluster 4 from this analysis as its small sample size could produce unreliable results in detecting admixture events.

### Selection signals

We applied the NSL statistic [21] to the three major clusters found in our dataset to understand how genetic structure affects the pattern of genes under putative positive selection. A conservative approach was considered, collecting only the results of markers previously associated with a phenotype, using, as a reference, the GWAS catalogue.

Scans for selection signals revealed that most hits are private to each cluster if we consider all the signals putatively functional (NSL >  = 99th percentile of the genomic distribution and presence in GWAS catalogue (see Figs. S6-S7).

Among the top signals that are shared between all three major clusters (NSL score over the 99th percentile and SNP present in GWAS catalogue), we found two variants in *the FADS2* gene: one rs174578, rs174583 and rs174601 associated with haemoglobin, serum metabolite measurement and different lipid traits (HDL, LDL), respectively [22–24]. Additional signals of shared selection signatures were found in *RYR1,* where the variant rs3786829 was associated with peanut allergy [25], another SNP was found in *DENND1A,* the variant carrying the signal (rs2479106) was associated with polycystic ovary syndrome [26]. Finally, we found selection signatures in three additional markers, one associated with microglial activation measurement (rs651691) [27], one associated response to anti-depressant treatment in major depressive disorder (rs10517287) [28] and one associated with trans-fatty acid levels (rs17099388) [29] (Table 1). Then we grouped the private signals of selection accordingly to the associated phenotypes. We discovered signatures of selection for genes linked to lipid traits, BMI and serum metabolite levels for Cluster 1 and Cluster 3. For Cluster 2, we found signals in SNPs involved in blood traits and height (Table S2).

**Table 1:** Shared signals of selection among the different subgroups in Qatar

SNP_id	Location	Consequence	SYMBOL	Gene	NSL Cluster1	NSL cluster2	NSL cluster3
rs651691	1:193,958,320–193,958,320	intergenic_variant	-	-	-3.42	-2.86	-2.66
rs10517287	4:33,624,702–33,624,702	intergenic_variant	-	-	-3.73	-3.12	-3.51
rs17099388	5:142,095,250–142,095,250	intergenic_variant	-	-	-3.36	-3.81	-3.15
rs2479106	9:126,525,212–126,525,212	intron_variant	DENND1A	ENSG00000119522	-2.83	-2.72	-3.04
rs174577	11:61,604,814–61,604,814	intron_variant	FADS2	ENSG00000134824	-2.95	-2.79	-3.14
rs174578	11:61,605,499–61,605,499	intron_variant	FADS2	ENSG00000134824	-3.63	-3.04	-3.19
rs174601	11:61,623,140–61,623,140	intron_variant	FADS2	ENSG00000134824	-3.96	-3.11	-3.63
rs3786829	19:39,014,184–39,014,184	intron_variant	RYR1	ENSG00000196218	-2.82	-3.79	-3.45

### Putative loss of function variation

Finally, we investigated how genetic structure affected the distribution and prevalence of loss of function variants. A total of 97 putative loss of function variants (LOF) were analyzed using a custom-made list described in the Method section. For thirty of them, a significant difference in frequency was found (after Bonferroni correction) only in one cluster compared to the others (see Table S3). The majority of them are specific to Cluster 1 (which shows higher homozygosity and Bedouin-like ancestry) and Cluster 2 (African ancestry). The markers with the highest difference in frequency in each cluster were then further analyzed (top five lowest p-values, corresponding to the top 2% of the results). One of them, rs2884737 (p-value = 5E-07), is located within *the VKORC1* gene and detected at high frequency in Cluster 1(Near Eastern ancestry). This variant is involved in warfarin response [30], A graphical representation of how ancestry determined the genotype distribution of these variants is shown in Figs. S6, S7 and S8. In Cluster 2(African ancestry), signals for rs1127745 located in *ACOX2* and associated with triglyceride levels [31]. One variant, rs35400274 (in *C17orf107*, a gene associated with Sphingomyelin levels [32], was present in Cluster 3 (South Asian ancestry). Finally, one variant, rs3213755, in *the KRTAP1-1* gene, which encodes for a keratin-associated protein, was found in Cluster 4; to our knowledge, there are no phenotypes previously associated with this gene. To investigate the relationship between effective population size and LOF distribution we applied the following approach: we grouped the LOF variants into two groups. The first one comprises high deleteriousness variants using CADD score [33] as measure of deleteriousness (CADD >  = 25), and the second one including low deleteriousness ones (CADD <  = 5); then, we estimated the median allele frequency in each group and each cluster found in the Qatari sample. The amount of low deleteriousness variation is related to the level of drift, and the amount of high deleteriousness variation indicates the natural selection efficiency. The ratio of high deleteriousness variation to low deleteriousness variation should hint at the efficiency of selection. A low ratio indicates higher purifying selection efficiency compared to drift. A high ratio suggests that selection is less efficient compared to genetic drift. As we can observe from Table S5 the lowest ratio is from Cluster 2 and the highest is from Cluster 1 which indicates that in the population with highest Ne, selection is more efficient.

## Discussion

Previously published works [5, 9, 34–36] described the different ancestral components in the Qatari population. Our focus is to describe how a peculiar genetic history shaped one population's genomic pattern in terms of homozygosity burden, variants under positive selection, and genetic drift of putative loss of function variants. With the current emphasis on precise and personalized medicine, and therefore on rare variants, we must not forget that demography and admixture shape the prevalence of common genetic factors that could impact the phenotype distribution at a population level, with repercussion on the welfare system.

With our findings, we provide a more comprehensive analysis regarding the ancestry-related structure that could be useful for future analyses on both array and whole-genome sequencing data (WGS). Three major ancestral groups (with predominantly Bedouin, African, and South Asian ancestry) named Cluster 1, Cluster 2 and Cluster 3 were found in agreement with previous data and uniparental marker analysis. The difference in variability between Y and mitochondrial data could hint at a sex-biased migration, in fact an higher haplotype variability in the mitochondrial genome respect to the Y chromosome could hint to movement of females in patrilocal groups [37]. Interestingly, a novel cluster with a small fraction of East Asian ancestry was found (Cluster 4), indicating additional cryptic gene flow from a more distant origin in the past. This additional cluster suggests that increased sample size could reveal higher levels of substructure than expected, further hinting at the Qatari population as a melting pot of different ancestries and admixture events [13]. Moreover, this scenario adds a new layer of complexity to the genetic architecture of the Qatari population. Therefore, for example, GWAS analysis should carefully consider this complex stratification to avoid any bias, for example, performing association studies in each ancestral subgroup separately, if possible, or selecting a method that can correctly take into account the cryptic structure of this and similar populations [38–40].

Our data showed how the population substructure is linked to the difference in ROH pattern, which affects phenotype distribution [6, 7, 41]. Cluster 1 showed higher levels of ROHs with respect to Cluster 2, Cluster 3 and Cluster 4, consequently. Overall, the present findings suggest a hierarchical level of population substructure in the Qatari population, characterized by varying levels of homozygosity. One limitation of our study is the lack of phenotype information. Despite some variants are found in homozygous state in a population, it is difficult to predict the overall variability of a phenotype linked to these markers, mainly because the majority of associated genetic variants explain very little of the phenotype variance.

Additional analyses revealed a different effective population size (Ne) between the three major clusters in recent time, such as the timing and number of admixture events. If we consider a generation time of 30 years, the time of the admixture events for cluster 1 is around 32 generations ago ~ 1040 CE (32 generations) while for Cluster 2 is ~ 1859 CE (5 generations). Cluster 3 shows two admixture events, one at 1859 CE (similar to cluster 2) and one at ~ 740 CE (42 generations ago). It is interesting how we can roughly overlap the admixture events for Cluster 1 and Cluster 3 to the period of the Abbasid Caliphate (750–1258 CE), where the Qatari region started to become a strategic economic hub, and pearl trading flourished. The most recent admixture events (for Cluster 3 and Cluster 2) correspond to the first stage in Qatar's development as a sheikhdom in recent history when the house of Thani started to rise in power [42]. Cluster 1 is the genetic group with lowest effective population and no evidence of recent admixture.

These results lead us to the assumption that also, the role of natural selection could be different. For this reason, we investigated the pattern of recent selection using nSL statistics. The analysis revealed that, despite all clusters sharing the same environment and actual geographical location, the selection signals are composed predominantly of private ones (~ 70%). These signals involve markers previously associated with lipid traits such as HDL and LDL (Cluster 1 and Cluster 3) and height and blood traits (Cluster 2).

Some of the signals are shared between clusters, such as variants in *FADS2*, which could be linked to diet adaptation [43, 44]. The pattern of shared signals is negatively correlated with the genetic distance between these three clusters. As previously shown, the selection pressure should come from an adaptation to a diet characterized by a high level of fatty acids derived from plants but relatively poor in fatty acids derived from fish or mammals [45] which could relate to the introduction of agriculture in the Middle East. One limitation in our analysis is that we based our assumptions on selection taking into account only specific variants reported in GWAS catalogue. Considering only a direct effect on a trait could restrict the possible explanations of selection pressures.

Besides signals of selection (related to ancestral origins), genetic drift shows different patterns in the Qatari population. Due to the reduced effective population size, we also expect reduced effectiveness of purifying selection. Thus, we investigated the pattern of a specific group of variants: the putative loss of function variants (pLOF). Our analysis revealed that there is a relative higher ratio of deleterious LOF (CADD >  = 25) in the clusters with lower Ne (Cluster1and Cluster 3), respect to the Cluster 2(Africans), which shows higher effective population size.

Our work showed that several common putative pLOF harbour significant differences in allele frequency between clusters. Some of them, like the variant in the VKORC*1* gene, are linked to a specific pharmacological response and show higher prevalence in Cluster 1 or are considered risk factors for phenotypes like triglyceride level ( *ACOX2* variant for Cluster 2).

The result on *VKORC1* is of particular interests, mainly because recent works showed the importance of warfarin management in the Qatar population [46] and how this gene is involved in warfarin dose variability in Qatari [47].Here we show that one genotype is more prevalent in one ancestry respect to another in the structured population of Qatar.

A study of the population structure of Qatar's people, as inferred by genetic testing, is necessary to determine how best to perform several association studies and other genetically-assisted analyses of risk in the Qatari population. Furthermore, our findings provide crucial information for risk stratification in the Qatari population.

## Material and methods

### Data preparation

Saliva samples from 188 healthy individuals were collected in Hamad Medical Corporation (HMC), A written informed consent for participation was obtained from all subjects. Samples DNA was extracted at the IRCCS Burlo Garofolo Hospital. Genotyping was conducted at the Life & Brain Research Centre (Bonn, Germany) using the Illumina Infinium Global Screening Array-24 v1.0 (GSAMD-24v1-0_20011747_A1). The initial quality control was performed on Illumina GenomeStudio software to remove poorly called samples and sites. Raw genotype data underwent a step of recalling using the software z-call [48] to obtain more reliable calls on low-frequency variants. PLINK v1.9 software [49] was used to process the genotype calls for further variants and samples QC: i) remove samples with high IBD sharing; ii) remove sites with a heterozygous rate higher than three standard deviations from the mean heterozygosity rate distribution; iii) remove sites and samples by call rate (–geno 0.01 –mind 0.05 options); iv) remove sites, not in Hardy–Weinberg equilibrium (–hwe 0.000001 option). The dataset resulting from these QC steps resulted in 186 individuals that was finally phased using the shapeit2 software [50], without using any reference panel.

### Y and mitochondrial haplogroup analysis

First, 28 male samples were extracted from the dataset and Y chromosome haplogroups were assigned using AMY-tree v2.0 software [51]. Input files were created by converting PED and FAM files into a vcf using PGDSpider v2.1.1.1 [52] and then from a vcf into AMY-tree input files with R scripts. Results were then combined using in-house R scripts. Mitochondrial analysis of 186 individuals was performed using the software haplogrep-2.1.20 [53]. Haplotype diversity was estimated following the formula described in [54]

### Population structure and admixture pattern

To obtain a larger picture of the geographical pattern we merged our dataset with 1000G Phase 3 [16] (dataset-A) and Human Origins dataset [55] (dataset-B). Principal component analyses on dataset-A and dataset-B were performed after removing markers in linkage disequilibrium using the option –indep-pairwise 200 50 0.4 implemented in PLINK [49]. Clustering approach was made using the R package Mclust [15] on the first 6 PCA eigenvectors. A complete list of all population used and their relative sample size is reported in Table S4.

Unsupervised admixture analysis using ADMIXTURE v1.23 [14] was done on dataset-B after removing the populations with less than ten individuals. Time of admixture using all possible combinations of reference populations was performed using MALDER [20].

Inbreeding and runs of homozygosity estimates were calculated using PLINK using the option –homozyg and –het.

We further investigated effective population size using IBDseq [56] and IBDNe [19] on each genetic cluster identified. We using a threshold of 2 centimorgan for IBD segments and default parameters as suggested for SNP array data.

### Selection scan

Selection scans in the different subgroups were done using the nSL statistic, a modification of iHS that has improved power in detecting soft sweeps [21]. Genotype data were phased using Eagle [57], and nSL statistics were estimated and normalized using selscan [58]. First, we collected the values with a score over than 2 and present in GWAS catalogue reported the fraction of private and shared variants under putative positive selection between the various subgroups. We then collected the results falling over the 99.9th percentile of the distribution of genomic nSL and we selected the variants reported in the GWAS catalogue. These analyses were done in order to assess the impact of natural selection in putatively functional variants already associated with disease or traits.

### Putative loss of function variant distribution

We created a manually curated dataset of LOF variants which was composed by two lists: the first set was a list of loss of function variants described in MacArthur et al. [59] while the second list was composed by all variants annotated as stop-gain using VEP tool [60]. This selection aimed to obtain a reliable list of putative loss of function variants. We grouped the LOF into two categories: one with CADD score >  = 25 which are considered as high deleterious and one with CADD score <  = 5, which are considered as low deleterious. We estimated the average allele frequency in each group in each genetic cluster.

For each LOF, using the function –assoc implemented in PLINK, we selected the differentiated ones on one cluster but not in the other. Only variants showing significant p-values after Bonferroni correction were further analyzed.

We investigated how ancestry affects the distribution of genotypes using the R package party [61], selecting the top differentiated markers in each subpopulation.

## Supplementary Information

**Additional file 1: Figure S1.** Distribution of Y and mitochondrial haplogroups in Qatari samples. **Figure S2.** Admixture runs from K=3 to K=11.At K=3 we can observe the separation between the Cluster 2 (with African ancestry) and the other three clusters, and we can observe the higher East Asian ancestry in Cluster 4. From K=5 to K=7 we can observe how the Bedouin ancestry is predominant in the Cluster 1 respect to the other clusters. At K=9 we can see how the South Asian (Sindhi)ancestry is becoming predominant in the Cluster 3. **Figure S3.** Principal component analysis.PC3 versus PC4 on the left panel, PC5 versus PC6 on the right panel. Variance explained by each axis is reported as well. **Figure S4.** Ancestry proportions in the Qatari sample. The red colour represents the Middle Eastern-Bedouin like ancestry, the cyan colour represents the South Asian component the violet component represents the Middle Eastern-Palestinian like component, the green one represents the African component and the blue one represents the East Asian component. **Figure S5.** Beanplot of Total Homozygosity in Qatar and 1000G populations. The dotted line represents the average worldwide level of total homozygosity. **Figure S6.** Genomic distribution of unstandardized —nSL— score. Each line represents the distribution of —nSL— values in each cluster, the dotted line represents the cut-off to discriminate between putatively under selection and neutral markers. **Figure S7.** Venn Diagram of shared and private signal of selection. The numbers represent the number of SNPs with nSL score over the 99th percentile of the genomic distribution and previously associated to a phenotype (GWAS catalogue) are reported. We can observe how the majority of the variants under selection and previously associated with a phenotype are private of each cluster and only a small fraction is shared between all of them. **Figure S8.** Regression tree analyses of rs2884737. The analysis shows the different and significant genotype distribution of rs2884737 among the four different cluster found in our dataset. The C allele in homozygous state is more prevalent in Cluster 1. **Figure S9.** Regression tree analyses of rs1127745. The analysis shows the different and significant genotype distribution of rs1127745 among the four different cluster found in our dataset. The G allele in homozygous state is more prevalent in the Cluster 2. **Figure S10.** Regression tree analyses of rs35400274. The analysis shows the different and significant genotype distribution of rs35400274 among the four different cluster found in our dataset. The A allele in homozygous state is more prevalent in the Cluster 1 and Cluster 2.

**Additional file 2: Table S1.** Cross validation errors from admixture runs.

**Additional file 3: Supplementary Table 1. **Phenotypes associated with private Signals of Selection among the different subgroups in Qatar.

**Additional file 4: Supplementary Table 2.** Signficantly differentisted pLOF variants between Qatari genetic clusters.

**Additional file 5: Supplementary Table 3. **Populations with relative sample size used in this study.

**Additional file 6: Table S5.** Level of diversity of low deleterious and high deleterious variants.

## Data Availability

A vcf file including all the variants with information on allele frequencies in the whole dataset, has been submitted to the European Variation Archive (EVA), study accession number: PRJEB51505. The data is accessible at the following link: https://www.ebi.ac.uk/ena/data/view/PRJEB51505**.** Data are also available upon request from the authors.

## Abbreviations

PCA: Principal component analysis
ROH: Runs of homozygosity
Ne: Effective population size
GWAS: Genome wide association study
nSL: Number of segregating sites by length
HDL: High-density lipoprotein
LDL: Low-density lipoprotein
BMI: Body mass index
pLOF: Putative loss of function
IBD: Identity by descent
QC: Quality control
