# Supplementary material for: Ancestry-related distribution of Runs of homozygosity and functional variants in Qatari population
Source: BMC Genom Data. 2022 Sep 21;23:73. doi: 10.1186/s12863-022-01087-1 (PMC9490902; doi:10.1186/s12863-022-01087-1)
Supplement: Supplementary file 1 — Additional file 1: Figure S1. Distribution of Y and mitochondrial haplogroups in Qatari samples. Figure S2. Admixture runs from K=3 to K=11.At K=3 we can observe the separation between the Cluster 2 (with African ancestry) and the other three clusters, and we can observe the higher East Asian ancestry in Cluster 4. From K=5 to K=7 we can observe how the Bedouin ancestry is predominant in the Cluster 1 respect to the other clusters. At K=9 we can see how the South Asian (Sindhi)ancestry is becoming predominant in the Cluster 3. Figure S3. Principal component analysis.PC3 versus PC4 on the left panel, PC5 versus PC6 on the right panel. Variance explained by each axis is reported as well. Figure S4. Ancestry proportions in the Qatari sample. The red colour represents the Middle Eastern-Bedouin like ancestry, the cyan colour represents the South Asian component the violet component represents the Middle Eastern-Palestinian like component, the green one represents the African component and the blue one represents the East Asian component. Figure S5. Beanplot of Total Homozygosity in Qatar and 1000G populations. The dotted line represents the average worldwide level of total homozygosity. Figure S6. Genomic distribution of unstandardized —nSL— score. Each line represents the distribution of —nSL— values in each cluster, the dotted line represents the cut-off to discriminate between putatively under selection and neutral markers. Figure S7. Venn Diagram of shared and private signal of selection. The numbers represent the number of SNPs with nSL score over the 99th percentile of the genomic distribution and previously associated to a phenotype (GWAS catalogue) are reported. We can observe how the majority of the variants under selection and previously associated with a phenotype are private of each cluster and only a small fraction is shared between all of them. Figure S8. Regression tree analyses of rs2884737. The analysis shows the different and significant genotype distr [file 12863_2022_1087_MOESM1_ESM.pdf]

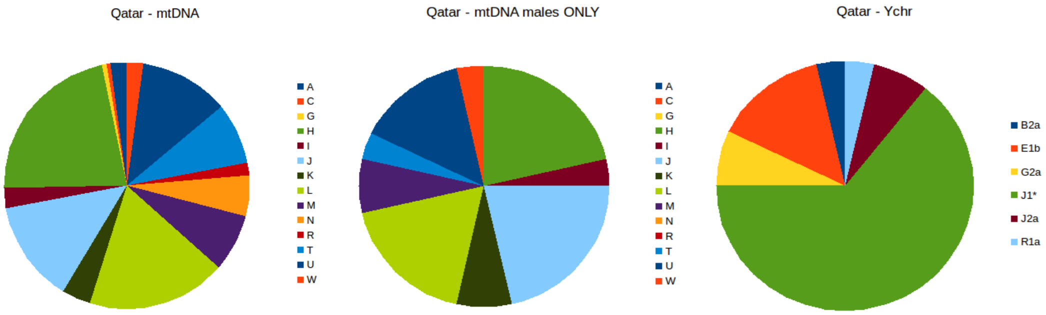

Figure (S1) **Distribution of Y and mitochondrial haplogroups in Qatari samples.**

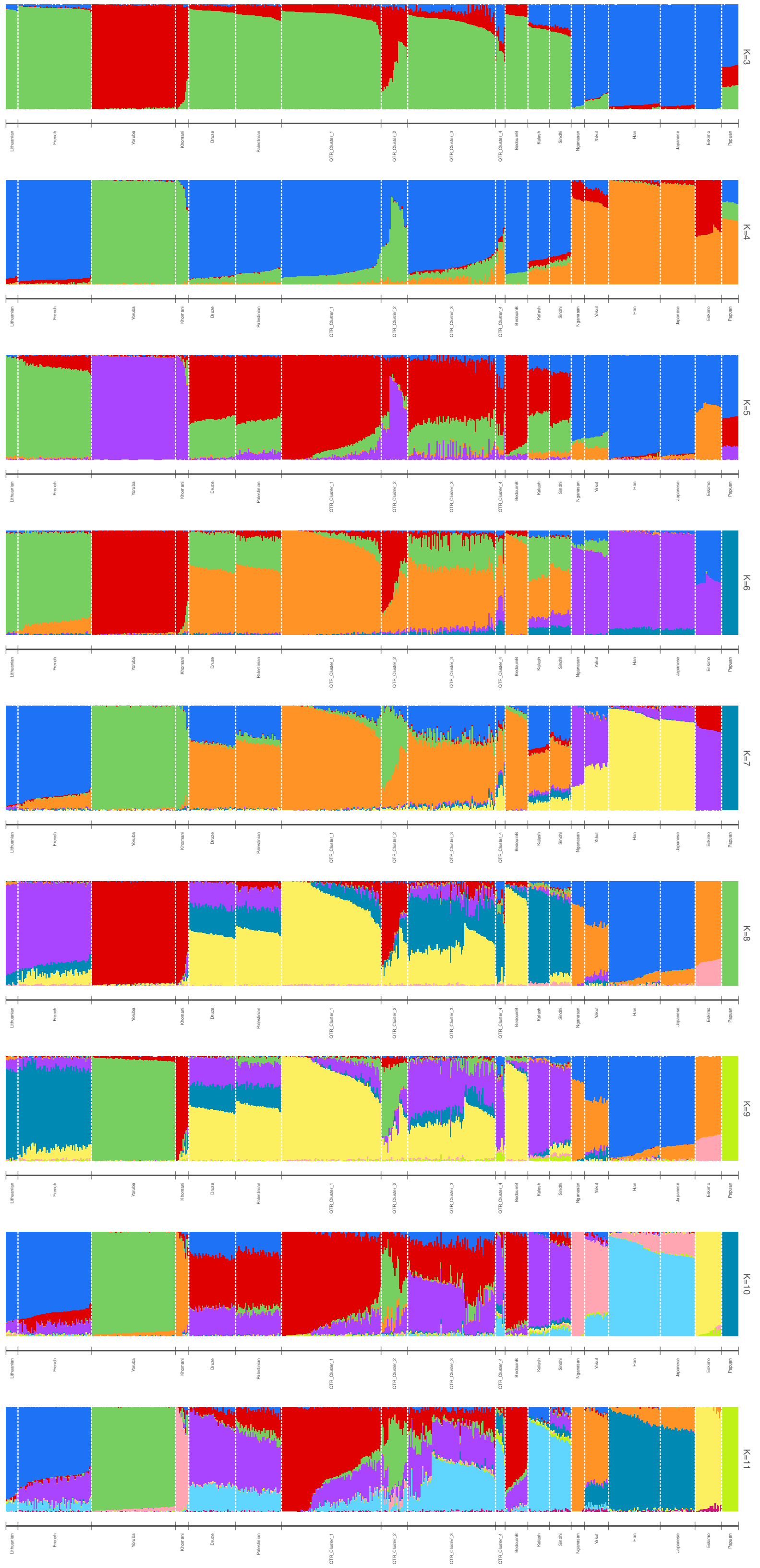

Figure (S2) **Admixture runs from K=3 to K=11.** At K=3 we can observe the separation between the Cluster 2 (with African ancestry) and the other three clusters, and we can observe the higher East Asian ancestry in Cluster 4. From K=5 to K=7 we can observe how the Bedouin ancestry is predominant in the Cluster 1 respect to the other clusters. At K=9 we can see how the South Asian (Sindhi) ancestry is becoming predominant in the Cluster 3.

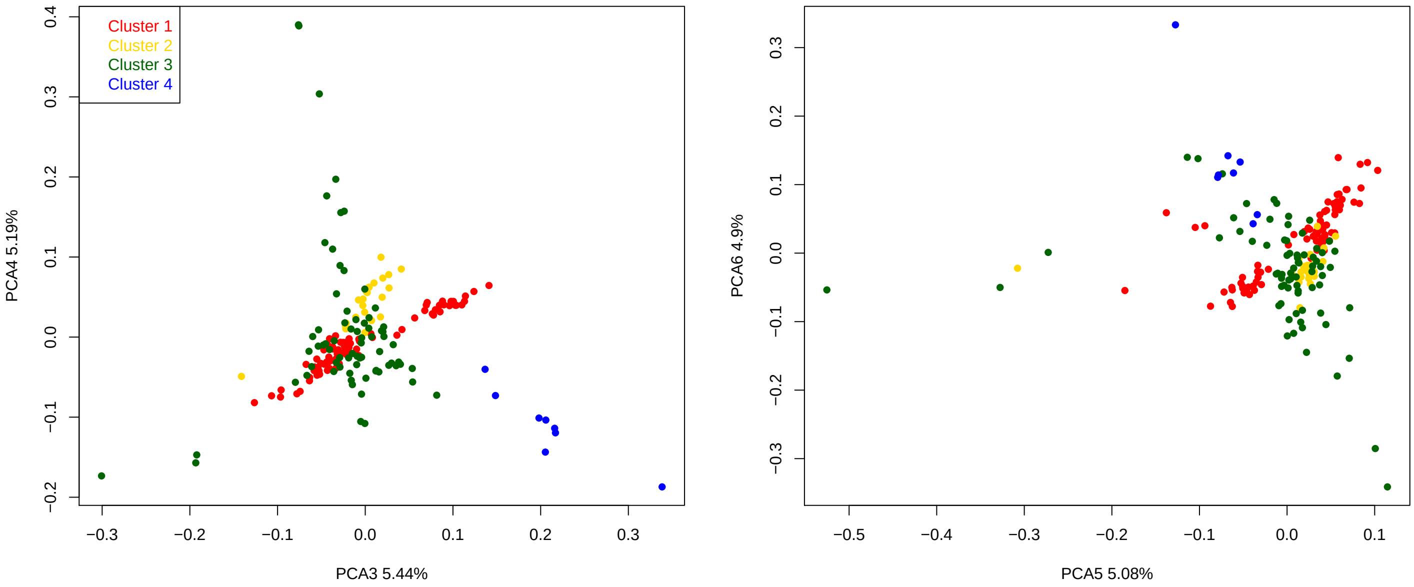

Figure (S3) **Principal component analysis.** PC3 versus PC4 on the left panel, PC5 versus PC6 on the right panel. Variance explained by each axis is reported as well.

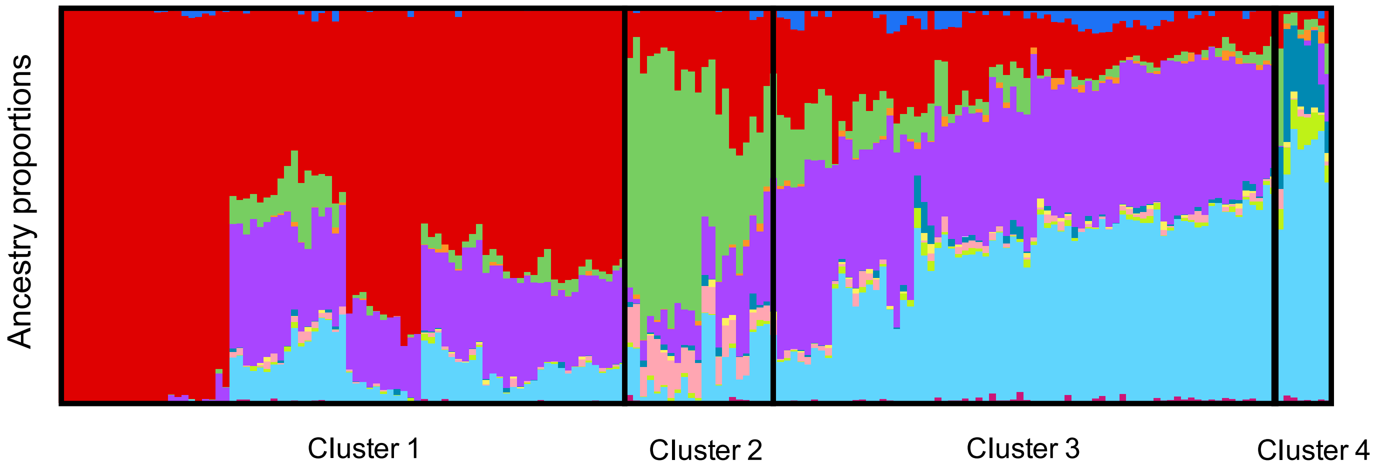

Figure (S4) **Ancestry proportions in the Qatari sample.** The red colour represents the Middle Eastern-Bedouin like ancestry, the cyan colour represents the South Asian component the violet component represents the Middle Eastern-Palestinian like component, the green one represents the African component and the blue one represents the East Asian component.

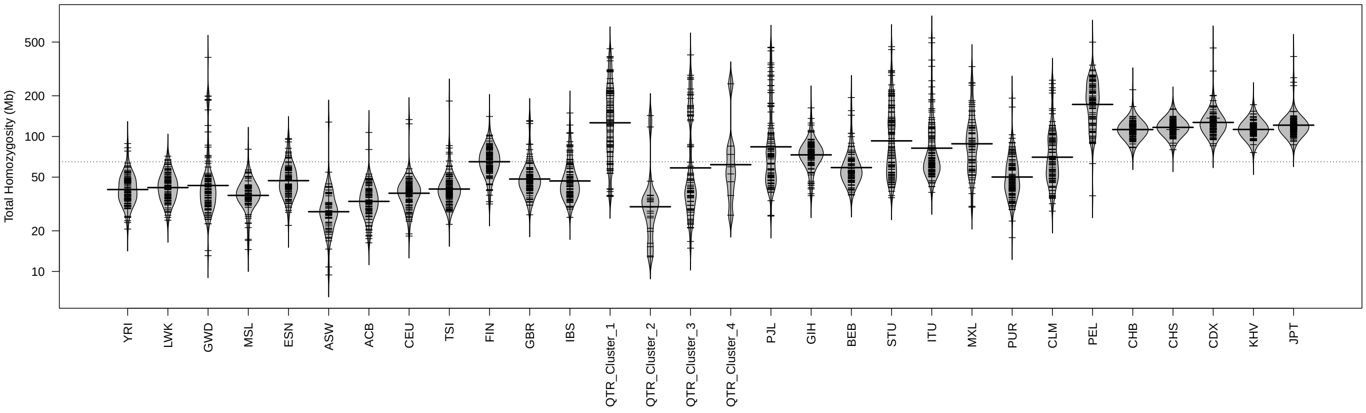

Figure (S5) **Beanplot of Total Homozygosity in Qatar and 1000G populations.** The dotted line represents the average worldwide level of total homozygosity.

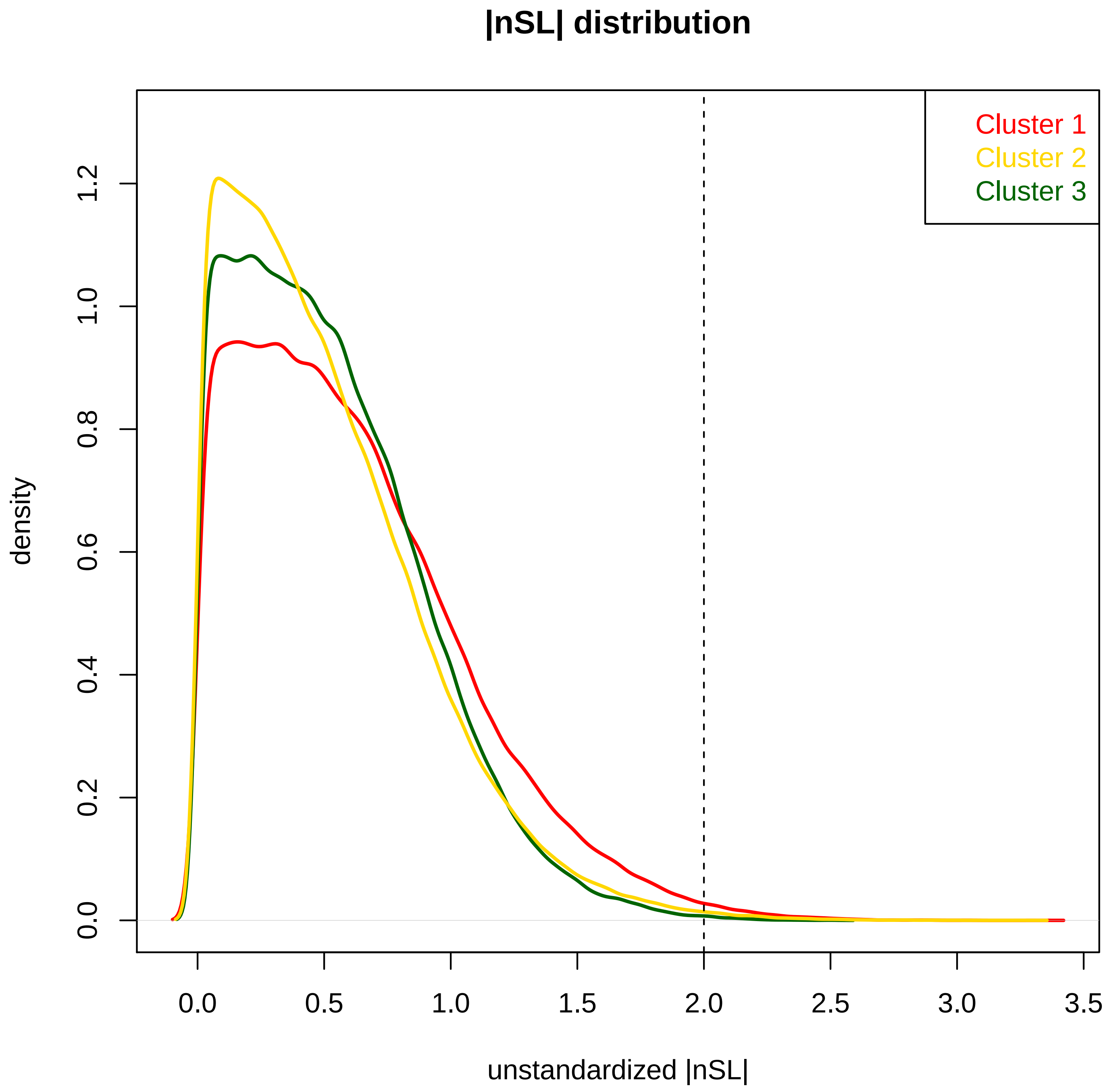

Figure (S6) **Genomic distribution of unstandardized —nSL— score.** Each line represents the distribution of —nSL— values in each cluster, the dotted line represents the cut-off to discriminate between putatively under selection and neutral markers.

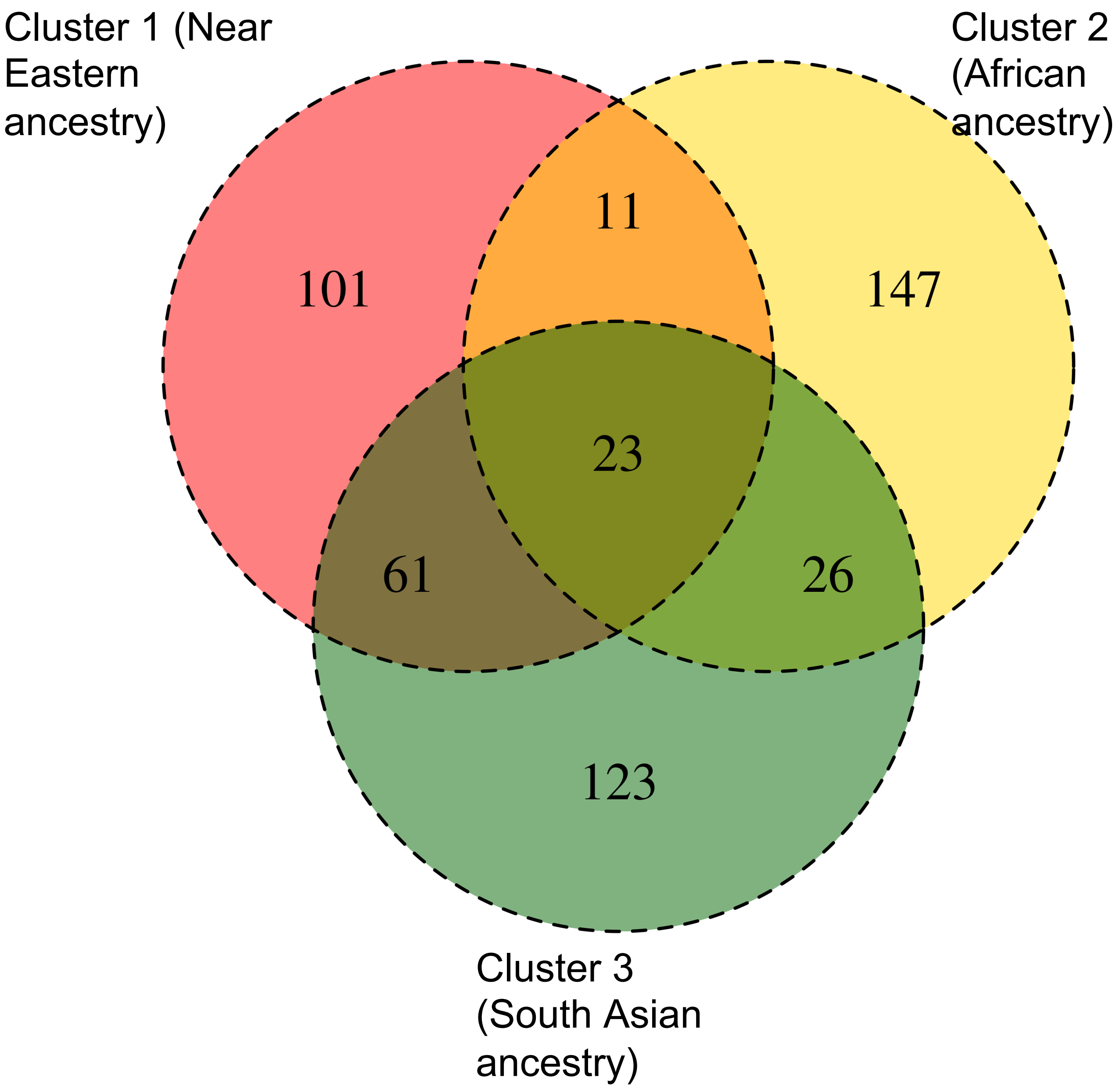

Figure (S7) **Venn Diagram of shared and private signal of selection.** The numbers represent the number of SNPs with nSL score over the 99th percentile of the genomic distribution and previously associated to a phenotype (GWAS catalogue) are reported. We can observe how the majority of the variants under selection and previously associated with a phenotype are private of each cluster and only a small fraction is shared between all of them.

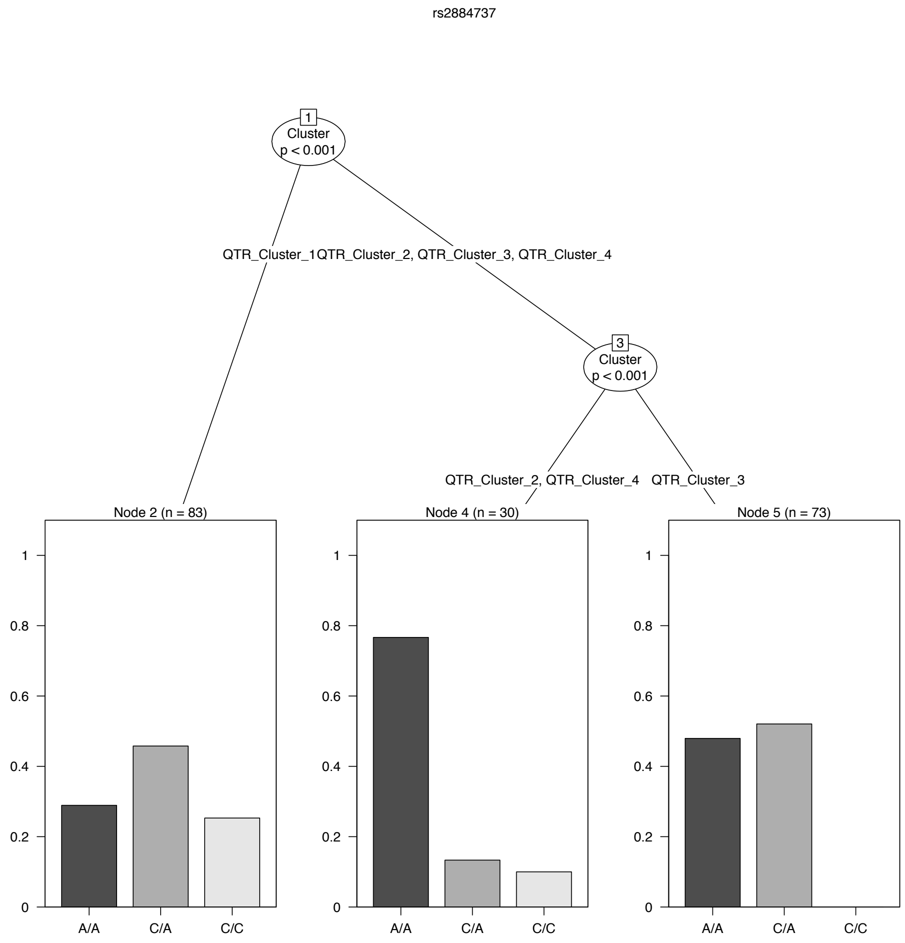

Figure (S8) **Regression tree analyses of rs2884737.** The analysis shows the different and significant genotype distribution of rs2884737 among the four different cluster found in our dataset. The C allele in homozygous state is more prevalent in Cluster 1.

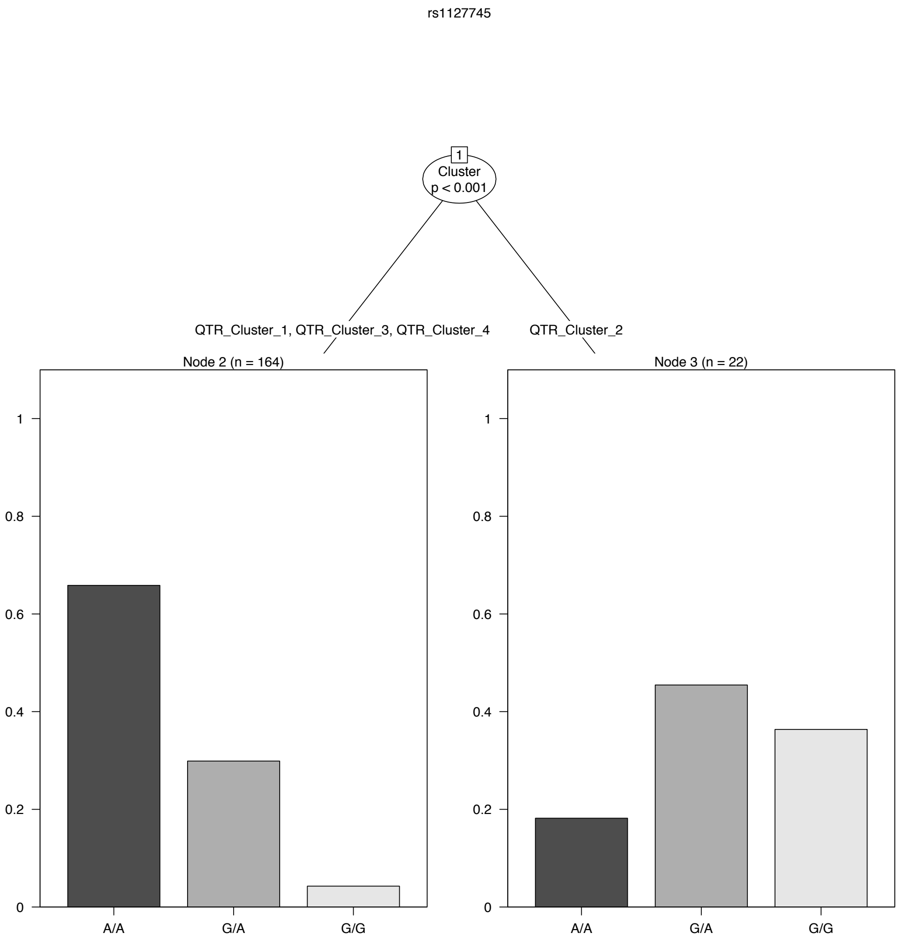

Figure (S9) **Regression tree analyses of rs1127745.** The analysis shows the different and significant genotype distribution of rs1127745 among the four different cluster found in our dataset. The G allele in homozygous state is more prevalent in the Cluster 2.

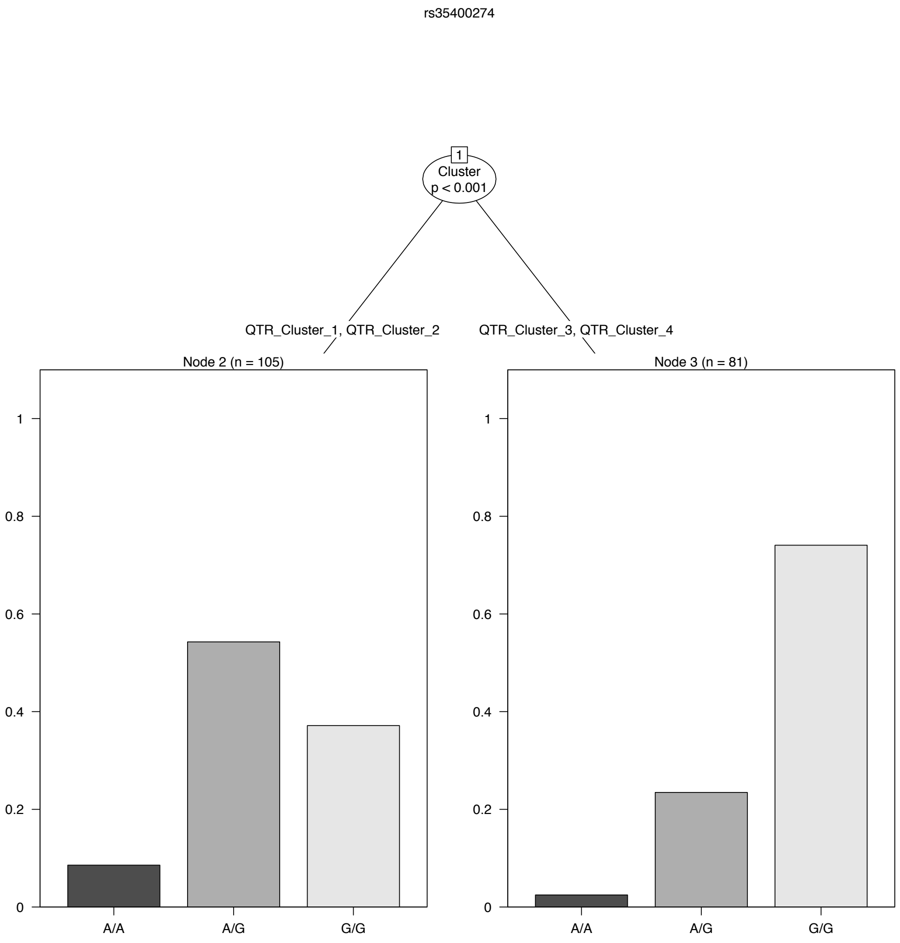

Figure (S10) **Regression tree analyses of rs35400274.** The analysis shows the different and significant genotype distribution of rs35400274 among the four different cluster found in our dataset. The A allele in homozygous state is more prevalent in the Cluster 1 and Cluster 2.
